# Frailty Screening Practice in Specialized Burn Care—A Retrospective Multicentre Cohort Study

**DOI:** 10.3390/ebj4010009

**Published:** 2023-02-13

**Authors:** Charlotte I. Cords, Cornelis H. van der Vlies, Matthea Stoop, Marianne K. Nieuwenhuis, Kris Boudestein, Francesco U. S. Mattace-Raso, Margriet E. van Baar, FRAIL Group, Dutch Burn Repository Group

**Affiliations:** 1 Association of Dutch Burn Centres, Maasstad Hospital, 3079 DZ Rotterdam, The Netherlands; 2 Trauma Research Unit, Department of Surgery, Erasmus MC, University Medical Centre Rotterdam, 3015 GD Rotterdam, The Netherlands; 3 Department of Trauma and Burn Surgery, Maasstad Hospital, 3079 DZ Rotterdam, The Netherlands; 4 Association of Dutch Burn Centres, Red Cross Hospital, 1942 LE Beverwijk, The Netherlands; 5 Association of Dutch Burn Centres, Martini Hospital, 9728 NT Groningen, The Netherlands; 6 Research Group Healthy Ageing, Allied Health Care and Nursing, Hanze University of Applied Sciences, 9747 AS Groningen, The Netherlands; 7 Department of Human Movement Sciences, University of Groningen, University Medical Center Groningen, 9713 AV Groningen, The Netherlands; 8 Department of Gerontology and Geriatrics, Maasstad Hospital, 3079 DZ Rotterdam, The Netherlands; 9 Section of Geriatric Medicine, Department of Internal Medicine, Erasmus MC, University Medical Center, 3015 GD Rotterdam, The Netherlands; 10 Department of Public Health, Erasmus MC, University Medical Centre, 3015 GD Rotterdam, The Netherlands

**Keywords:** burn care, elderly, frailty screening, feasibility, Dutch Safety Management Program, validity

## Abstract

*Background:* Frailty can have a negative influence on outcomes in elderly patients after burn injuries. The Dutch hospitals have used a four-domain frailty screening instrument from the Dutch Safety Management System (DSMS) since 2012. However, its feasibility and validity have hardly been studied. We aim to assess the feasibility and validity of frailty screening in specialized burn care. *Methods:* A multicentre retrospective cohort study was conducted in all Dutch burn centres. Patients aged ≥ 70, with a primary admission between 2012–2018, were included. Data were derived from electronic patient files. *Results:* In total, 515 patients were included. Frailty screening was complete in 39.6% and partially complete in 23.9%. Determinants for a complete screening were admission after 2015 (OR = 2.15, 95% CI 1.42–3.25) and lower percentage TBSA burned (OR = 0.12, 95% CI 0.05–029). In all completely screened patients, 49.9% were at risk of frailty. At risk patients were older, had more comorbidities (known group validity), a longer length of stay, and more frequently a non-home discharge (predictive validity). *Conclusion:* Frailty screening in specialized burn care is feasible and was conducted in 63.5% of admitted patients. In total, 44% of screened patients were at risk of frailty. Validity of frailty screening was confirmed. Frailty screening can contribute to optimal specialized burn care.

## 1. Background

Because of an ageing population worldwide, an increase in the need for hospital care for the elderly is foreseen in the coming years [1]. As the elderly are particularly susceptible to burn injuries, hospital admissions in specialized burn care facilities are expected to increase accordingly [2]. Despite an increase in the overall quality of care in specialized burn centres over the years, elderly patients are at risk of being frail and, therefore, with other factors, such as comorbidity and polypharmacy, are at risk of adverse outcomes, such as falls, an increased number of complications, rehospitalisation, care dependency and mortality [3]. To support decision-making for prevention and to decrease health risk as much as possible, the identification of frail patients is essential [4,5,6]. Frailty is characterized by a decrease in reserves and resistance to stressors caused by declines in physiologic systems that can result in increased vulnerability [7,8].

Dutch hospitals, including Dutch specialized burn care, are obliged to screen all patients aged ≥ 70 with the Dutch Safety Management System (DSMS frailty screening) [1,9]. The DSMS frailty screening is based on four geriatric domains: risk of falls, delirium, ADL dependence and malnutrition [7,9]. Only a few publications report on frailty assessment in burn care, predominantly based on research data [10,11,12,13]. Little is known about the feasibility of the DSMS frailty screening in an acute setting, even though feasibility assessment is a crucial part of determining the efficacy and usefulness of a screening intervention [14,15]. The predictive validity of the DSMS has been previously described, but not in a burn care setting [5,6,16]. Other aspects of validity have not been assessed yet.

To learn more about the feasibility and validity of DSMS frailty screening, a critical evaluation of current screening practice is needed. The primary aim of this study was to assess the feasibility of DSMS frailty screening in specialized burn care. Predictors for complete screening were also assessed. Additionally, the prevalence of frailty was assessed. Finally, the known group validity and predictive validity of the DSMS frailty screening were assessed. We hypothesized that patients at risk of frailty would be older and have more comorbidities (known group validity). In addition, we hypothesized that patients at risk of frailty would have a worse outcome, including longer length of stay, and more frequent non-home discharge (predictive validity).

## 2. Methods

### 2.1. Study Design and Participants

In this multicentre observational retrospective cohort study, all patients aged 70 years and over with a primary admission to one of the three Dutch burn centres (Maasstad Hospital Rotterdam, Martini Hospital Groningen and Red Cross Hospital Beverwijk) between 1 January 2012 and 31 December 2018 were eligible for inclusion. Burn centre care in the Netherlands includes not only burn injuries, but also patients with other skin-related diseases needing advanced care, with around ten admissions a year in all burn centres combined [17]. The local institutional review board of the three hospitals approved the study protocol (L2019095); the law on medical research with humans was not applicable according to the Medical Ethics Committee, MEC-U (W19.205).

### 2.2. Frailty Assessment 

**Figure 1:**
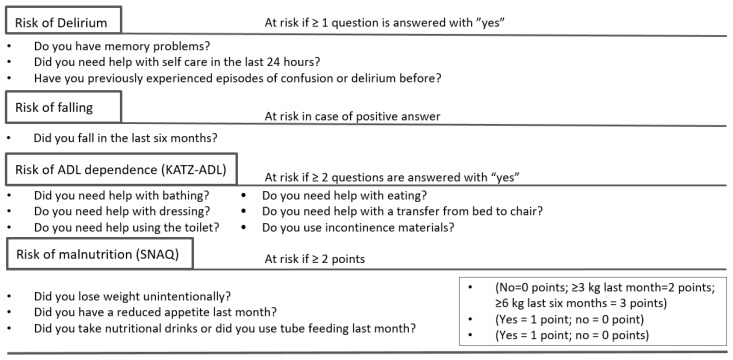
Dutch National Safety Management System, frailty screening (DSMS). KATZ-ADL = Katz index of independence in activities of daily living; SNAQ = Short nutritional assessment questionnaire.

Frailty was assessed with the DSMS frailty screening, consisting of four screening domains: risk of delirium, falling, ADL dependence and malnutrition (Figure 1) [9]. The tool is freely available, takes four to five minutes to fill in, and is usually filled in by a treating nurse [18,19]. The risk of delirium is assessed with three questions. When at least one of three questions is answered with ‘yes’, this suggests an increased risk of delirium [9]. The risk of falling is assessed by one question and, if answered with ‘yes’, the patient is considered at risk of falling. Screening for ADL is assessed through the six-item Katz Index [20]. When at least two questions are answered with ‘yes’, the patient is considered at risk of ADL dependence. Risk for malnutrition is assessed using the three-item Short Nutritional Assessment Questionnaire for Residential Care (SNAQ) score. A score of ≥2 suggests a higher risk of malnutrition [21]. The questions are administered by a nurse within 24 h of admission and answered with ‘yes’ or ‘no’ by the patient or their proxy. A cumulative frailty screening score (0–4) can be calculated. The more domains are scored positive, the higher the frailty risk. As well as this ordinal scale, the DSMS can be used in a dichotomized manner where patients are ‘at risk of frailty’ if two or more of the four frailty screening domains are scored positive. Heim et al. assessed how many domains should be positive for the most reliable frailty diagnosis. They found that two or more domains were most predictive of adverse outcomes (Figure 1) [1,5,6,9,16,22].We use this dichotomized method of frailty assessment to assess validity. In terms of feasibility, the DSMS frailty screening was reported as completed if all four domains were filled in entirely. The separate domains were considered complete if all questions were filled in. The primary study outcome was a complete screening of the DSMS frailty screening (yes/no).

### 2.3. Data Collection

Data on DSMS frailty screening, patient characteristics, burn characteristics, treatment characteristics and outcomes were documented (Table 1). Feasibility was assessed, looking at the percentage of completed screening assessments, the number of domains screened and possible trends in completely filled-in assessments over the years. Data on DSMS frailty screening were derived from the electronic patient files by a member of one of the local research teams. Comorbidity, according to the American Society of Anaesthesiologists (ASA) score, was derived or calculated by a research team member if not available from the electronic patient files. Data on patient characteristics, burn injury, treatment and outcome (in-hospital mortality, discharge location) were derived from the national registry in Dutch specialized burn care (Dutch Burn Repository R3), which started collecting data from 2009 onwards. The NBR R3 is filled by dedicated burn care professionals, with a coordinator who formally organizes quality monitoring and improvement.

### 2.4. Data Analysis and Statistical Analysis 

**Figure 2:**
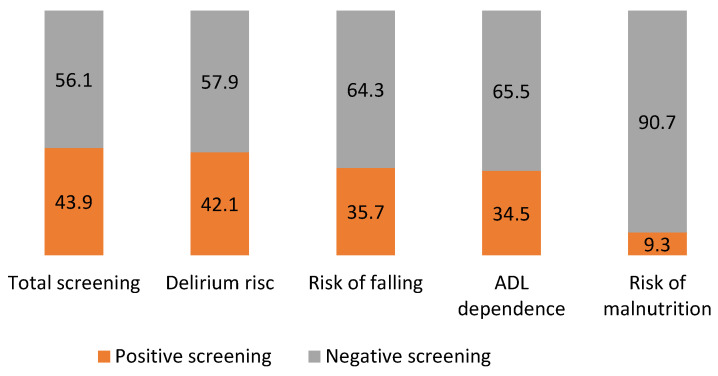
Prevalence of positive DSMS frailty screening domains.

To assess the feasibility of the DSMS frailty screening, we analysed the percentage of completed screening assessments, the number of domains screened and the possible trend in completely filled-in assessments over the years. Furthermore, possible predictors for a complete DSMS frailty screening were examined using logistic regression analysis adjusted for possible confounders (i.e., sex, TBSA and inhalation injury). Outcomes were reported as percentages for categorical variables, and continuous variables were summarized as either means with corresponding standard deviations (SD) or medians with 25th –75th percentiles depending on the normality of distribution. Relative risks were estimated by odds ratios (ORs) with 95% confidence intervals (CIs). To examine factors related to complete screening, univariable and multivariable regression (forward stepwise LR) analyses were performed, as shown in Table 2. A *p*-value of <0.20 from the univariable regression analyses was considered to reflect a relationship between a variable and positive screening. In the multivariable analyses, variables were checked for multi-collinearity (Spearman’s r > 0.75). Multivariable analyses were performed with a minimum of 10 cases for every estimated parameter. The prevalence of risk of frailty and the risk of the individual domains were assessed and scored as complete positive screening ‘yes’ or ‘no’ (Figure 2).

The known group validity was tested. It was hypothesized that DSMS frailty screening outcome on admission could differentiate between patient groups with different age and comorbidity levels. Older patients (>75 years) and patients with severe comorbidity (ASA ≥ 3) were observed to be more likely to be frail using logistic regression (Table 3). Furthermore, the predictive validity of the DSMS frailty screening was tested. It was hypothesized that a positive frailty screening on admission (DSMS frailty score ≥ 2) would be related to adverse outcomes, including increased length of stay, higher in-hospital mortality rates, and non-home discharge location in the elderly burn centre population, using logistic regression analysis adjusting for age, TBSA and inhalation injury (Table 4). Sensitivity analyses were conducted to examine the results for known group validity and predictive validity in patients with burn injury only (excluding patients with other skin-related diseases) (Appendix A). To analyze the association between a positive frailty score and determinants and adverse outcomes, we first used univariable logistic regression analysis (Table 5). Next, we conducted exploratory analyses, to study the effects of age, sex, ASA score, burn size, inhalation injury and a positive DSMS score (≥2) on length of stay, discharge location and in-hospital mortality (Table 6). Data were analyzed using IBM SPSS Statistics for Windows version 26 (IBM Corp., Armonk, NY, USA).

## 3. Results Inclusion

In total, 515 patients aged ≥ 70 were admitted to a Dutch burn centre between 2012 and 2018. Of these patients, 50.5% were women, the median age was 78.8 (25th–75th percentiles 73.0–84.0) and the median TBSA burned was 4.0% (25th–75th percentiles 1.5–10.0%). The majority of admitted patients (84.2%) had burn injuries. Table 1 shows an overview of all patient and injury characteristics.

### 3.1. Feasibility of the DSMS Frailty Screening

**Figure 3:**
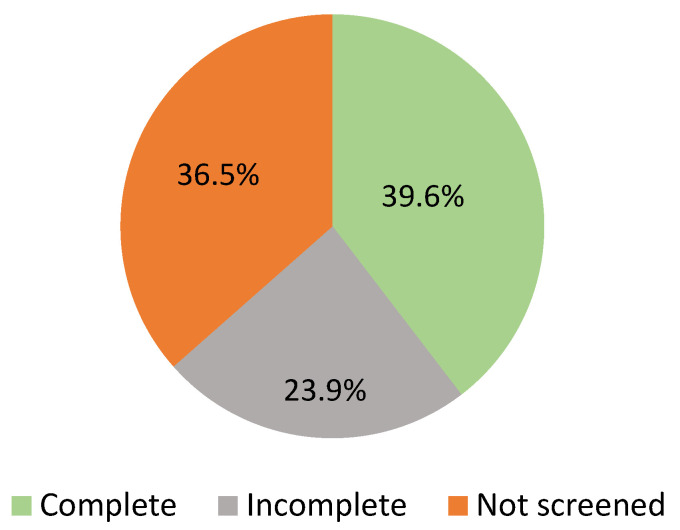
DSMS frailty screening practice (*n* = 515).

The DSMS frailty screening was complete in 39.6% of patients. A partially complete screening was seen in 23.9%. Of all included patients, 11.7% were screened on three domains. Screening on two domains and one domain was seen in 6.0% and 6.2% of patients, respectively. Approximately one-third of all patients (36.5%) were not screened, or screening was not documented (Figure 3). Screening on the risk of malnutrition was completed in most cases, while the risk of falling was filled in the poorest (Table 7).

### 3.2. Determinants of Complete Frailty Screening 

**Figure 4:**
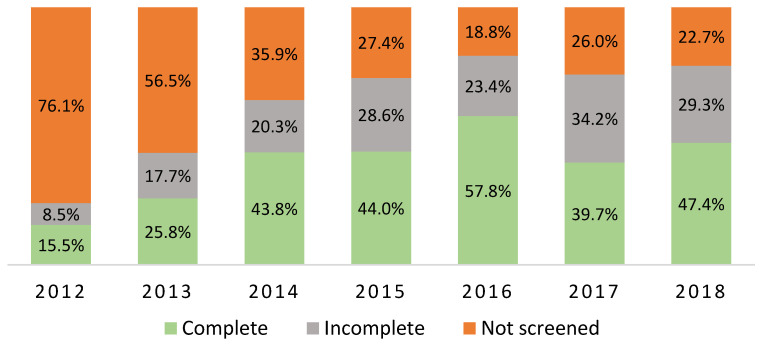
Screening practice per year (*n* = 515 patients).

As Figure 4 demonstrates, an increase in complete screening was seen over the first few years, with a slight decrease after 2016. Screening practice did not differ by patient diagnosis—40.5% completed screening in patients with burn injuries versus 35.7% in patients with other skin-related diseases (*p* = 0.53). The characteristics of patients with a complete screening versus an incomplete or missing screening are shown in Table 2. Completely screened patients were more likely to be admitted after 2015 (OR 1.89, 95% CI 1.32–2.70). They were less likely to be admitted to the intensive care unit (ICU) (OR 0.44, 95% CI 0.29–0.68), were more likely to have <20% TBSA burned (OR 0.19 CI 95% 0.09–0.42) and had a lower revised Baux score (OR 0.98 CI 95% 0.96–0.995) compared to patients that were not (completely) screened. Multivariate regression revealed two independent predictors for complete screening, including admission after 2015 and a lower percentage of TBSA burned (<20% TBSA).

### 3.3. Risk of Frailty 

According to the DSMS frailty screening, 141/228 (61.8%) scored positive on at least one frailty domain and 100/228 patients (43.9%) had a positive frailty screening (≥2 DSMS frailty screening domains scored positive), indicating an increased risk of frailty. Risk for delirium scored positive (42.1%) most frequently, while screening on risk of malnutrition was positive in a minority of patients (9.3%). Figure 2 shows an overview of the screening results.

### 3.4. Validity of the DSMS Frailty Screening: Known Group Validity and Predictive Validity 

As hypothesized, patients with a positive frailty screening were more likely to be older (OR 2.85, 95% CI 1.58–5.12) and had a higher ASA score, reflecting more severe comorbidity (OR 3.91, 95% CI 2.24–6.82) at admission. The hypothesis concerning the predictive ability of adverse outcomes of the DSMS frailty screening was correct for two out of three adverse outcomes: a positive frailty screening was predictive of a longer length of stay (OR 1.97 95% CI 1.05–3.70) and a non-home discharge location (OR 3.20 95% CI 1.69–6.12). After correcting for illness severity (age, TBSA and inhalation injury), DSMS frailty screening was not predictive of in-hospital mortality (OR 3.66 95% CI 0.68–19.86). Sensitivity analysis in patients with burn injury only showed similar results for known group and predictive validity (excluding patients with other skin-related diseases). Exploratory analyses were conducted to assess the predictive validity of separate DSMS frailty domains on adverse outcomes. Positive risk on delirium predicted length of stay (OR 1.80, 95% CI 1.02–3.16) and discharge location (OR 2.39, 95% CI 1.38–4.15). Positive risk of falling predicted length of stay (OR 2.20, 95% CI 1.15–4.20), discharge location (3.28, 95% CI 1.79–5.98) and in-hospital mortality (OR 3.88, 95% CI 1.08–13.96). Risk of ADL dependence predicted discharge location (OR 3.33, 95% CI 1.91–5.81). Risk of malnutrition was not predictive of adverse outcomes. Additional analyses were conducted to explore the association between a positive frailty score, patient characteristics, injury characteristics, and adverse outcomes. Table 5 shows a significant association between positive frailty score (DSMS ≥ 2) and higher age and more severe comorbidity (ASA score > 2). Furthermore, it shows a significant association between a positive frailty score and a longer (>7 days) length of stay, a discharge location other than home, and in-hospital mortality. In multivariate analyses, the female sex and a positive frailty score were associated with longer length of stay. Burn size, more severe comorbidity (ASA > 2), and a positive frailty score were associated with a non-home discharge. Finally, age and burn size, but not frailty, were associated with in-hospital mortality (Table 6).

## 4. Discussion

This multicentre study showed that 63.4% of patients were screened using the DSMS frailty screening instrument. Screening was most often completed in patients admitted after 2015 and in less severely injured patients. The feasibility seems adequate, except in patients with severe burns. In total, 43.9% had a risk of frailty in two or more domains and more than 60% scored positive on at least one frailty domain. Delirium was the domain most frequently screened positive. Both known group validity and predictive validity were shown by hypothesis testing. A positive frailty screening result was seen more frequently in older patients and in patients with severe comorbidity. In addition, frail patients had a longer length of stay and were more likely to be discharged to a non-home location.

This study showed that DSMS frailty screening was carried out in 63.4% of patients (including 39.6% completely screened patients, and 23.8% partially screened patients). No other studies are available on the feasibility and validity of the DSMS frailty screening in burn care. Other studies, including our systematic review, have reported on the completion rate of several frailty screening tools in an acute trauma setting. They found results in line with our study, with 48–71% complete frailty screening rates [18,23,24]. In these studies, clinicians filled in the screening [24,25,26,27]. Higher completion rates up to 100% were only seen in studies where screening was conducted by a research team [1,11,12,13,28,29,30,31]. This difference might be explained by the lack of time available for clinicians and the high workload in a busy acute care hospital setting. Clinicians might feel less urgency to complete the screening instrument, especially when a patient appears to be healthy and fit, or is very ill and admitted to the ICU. The support and assistance of nurses and other health care professionals are essential, since they are the ones who fill in the assessment and, most importantly, the ones to undertake the next step after a positive screening. If they do not experience added value, it is most likely that the assessment will not be performed. A recent study showed that nurses think frailty screening instruments can be helpful in daily practice, but judge their own clinical view as more important [14]. It is possible that more knowledge on the risks of frailty and its possible consequences would underline the value of frailty screening among those who carry out the screening.

Two other frailty screening instruments have been used in a burn care setting, but only in a research context [10,11,12,32]. The “Clinical Frailty Scale” (CFS) has been used in two retrospective studies, where it was predictive of morbidity, in-hospital mortality and one-year mortality, but its feasibility [11,13]. The CFS is a scale with nine steps, ranging from one, “very fit”, to nine, “terminally ill”. In contrast to the DSMS frailty screening, the CFS has no subdomains [33]. It is reported to take a minute to fill in and is intended to be filled in by the treating physician [18,34]. In recent literature reports, the Clinical Frailty Scale (CFS) has been used frequently in ICU settings that might be comparable to a burn care setting. Its feasibility was demonstrated by a study where the CFS was completed in 99.8% of the included 5187 patients [35,36]. Furthermore, our recent systematic review on frailty assessment in acute trauma patients showed that the CFS appears to be a feasible tool for use in an acute care setting in general [18].

The “Burn Frailty Index” (BFI) is the second frailty assessment tool that has been described in burn care. It has been shown to be predictive of morbidity and mortality in patients with burn injuries; however, the authors did not report on its feasibility [32]. The BFI contains physical and cognitive subdomains and includes 15 questions that can be asked of the patient or their proxy [32]. A treating physician should fill in the BFI; the time needed to fill in the tool has not been described [32].

This study showed that DSMS frailty screening was more prevalent after 2015. This increase in screening practice over the years is in line with what can be expected; it takes time for a process to become routine. So, this increase in screening assessments over the years represents the learning curve of the nurses who perform the screening. A previous literature report stated that routine practice of DSMS frailty assessment only started from 2015 onwards in the hospitals included in the study [23].

The fact that lower TBSA burned, lower revised Baux score, and admission to a regular ward instead of the ICU, are all (univariate) predictors for a complete DSMS frailty screening suggests that illness severity has a significant influence on screening behaviour and might indicate that frailty screening using the DSMS is less feasible for severely injured patients. It is probable that a less extensive instrument, for instance, the CFS, would result in a more complete screening procedure for more severely injured patients. To our knowledge, no study has been conducted assessing DSMS frailty screening in an ICU setting.

Complete screening of the separate frailty domains was also assessed in this study, with completion rate varying between the domains. The risk of falling had the lowest completion rate (49.5%), while risk of malnutrition had the highest completion rate (55.5%). This high screening rate might be explained by the fact that, in specialized burn care, it is common practice to consult a dietician almost immediately after admission, resulting in optimal screening practice on this domain.

In most cases, a positive DSMS frailty screening result was based on positive screening for the risk of delirium; a positive screening for risk of delirium was seen in most patients (42.1%). Thus, delirium seems to be an important frailty factor in our care setting that is also actively considered in our treatment. As van Yperen et al. stated, delirium in burn care is strongly correlated with longer hospital stay and in-hospital mortality [37]. Other studies found a relationship between frailty and delirium [38,39]. Being delirious might, thus, be a predictor for frailty and should be examined in future studies. The risk of malnutrition had the lowest positive screening score of 9.3%. This was also lower than that seen in previous literature reports. A study on geriatric inpatients in a Dutch hospital showed positive screening for risk of malnutrition in 39% of patients [1]. A study conducted in an emergency department showed a higher risk of malnutrition of 67% for frail patients [16]. In burn patients, malnutrition might be seen less often at admission, but, if present, is still predictive of frailty in patients in dedicated burn care and should, thus, be taken into account in every patient. In addition, it is important to keep in mind that patients with burn injuries, or other skin-related diseases, are at risk of developing malnutrition during the hospital stay due to the loss of fluids and a hyper-metabolic response as a result of their injury [40].

The DSMS frailty screening was originally developed to assess the four individual frailty domains to prevent adverse outcomes for every domain. Subsequently, patients were divided into frail (two of more DSMS domains positive) and non-frail (less than two DSMS domains positive) patients in some studies [5,9,14]. This method has proven value, but might lead to the loss of valuable information, especially as this study showed that more than half of the patient population scored positive on one or more frailty domains [6]. This study and that of Oud et al. showed that the individual domains of the DSMS can interact, underlining the importance of examining each of the four different domains on their own, since the needs of the elderly might vary depending on which domain(s) are scored positive [6]. In addition, personal interventions could be started that focus on the domains that need attention. To date, it is unclear to what extent preventive measures are being implemented as standard care after positive screening. It is, therefore, essential to explore this in the future. Since the DSMS can be predictive of poor outcomes, and its separate domains can be of great importance, we believe that optimal education on interpretation and training on implementation and execution of the screening should be encouraged in specialized burn care.

In this study population, risk of frailty according to the DSMS frailty assessment was present in one out of five screened patients. No other studies assessing frailty using the DSMS frailty screening are available on patients with burn injuries. There are, however, three studies that showed higher percentages of patients at risk of frailty following DSMS frailty screening, ranging between 25% and 68%. However, these studies were conducted in different settings, including patients admitted to the emergency department and screened after hip surgery [5,16,24].

Exploratory analyses were conducted to study the association between a positive frailty score using the DSMS and patient characteristics, injury characteristics and adverse outcomes. Univariable logistic regression showed a relation between adverse outcomes, including in-hospital mortality, and a positive frailty score. After adjustments, however, the DSMS was not predictive of in-hospital mortality. Further analyses are necessary to assess the possible relation between frailty and mortality and other adverse outcomes, preferably with data from a large prospective cohort study.

Our data showed the known group validity and predictive validity of the DSMS frailty screening in specialized burn care. Patients at risk of frailty were older, had more comorbidities, had a longer hospital stay and more frequent non-home discharge disposition. These results were as hypothesized and in line with the previous literature on this subject [3,11,13]. This study did not confirm the expected relation between risk of frailty and increased in-hospital mortality, which was seen in previous studies [3,6,11,13,24]. Several studies have been conducted on the predictive validity of the DSMS frailty screening, but none were conducted in specialized burn care in particular [1,16,24].

### Strengths and Limitations

This multicentre study provides a detailed overview of the current frailty screening practice in specialized burn care, including related skin-disease admissions. Feasibility is one of the most essential clinical requirements for a screening instrument, and this study is, therefore, a valuable addition to the literature. There are, however, some limitations. The retrospective nature of this study uses only available data, and lacks additional information that might be of use but was not documented in the electronic patient file. For instance, the reason for missing screenings of patients remains unknown. It is possible that the screening was not documented after careful consideration by the treating nurse, or screening was negative and documentation was considered unnecessary. Furthermore, qualitative interview or prospective observations on feasibility would have been a valuable addition but were not feasible due to the retrospective design of this study. We recommend assessing qualitative research on this topic in the future. The DSMS frailty screening is a Dutch screening instrument and, thus, our results do not directly apply to other multidomain frailty scores. However, since other frailty assessments with comparable items are very similar, the feasibility results can be expected to be reasonably similar. Finally, we included all patients admitted to our burn centres, including patients with other skin-related disease. The feasibility of frailty screening seemed similar. We refrained from further analysis within this subgroup because of a limited number of patients in the non-burn patient group. 

## 5. Conclusions

This study provides an insight into the feasibility of current frailty screening practice in specialized burn care and in relation to several aspects of validity. The relevance of frailty screening in burn care is shown by the elevated risk of frailty in 43.9% of screened patients. A substantial proportion of patients admitted to specialized burn care were screened. As hypothesized, the screening instrument could distinguish between known groups of patients based on age and comorbidity status and was predictive of adverse outcomes for frail elderly patients. However, to improve overall screening practice, optimal education on interpretation and training on implementation and execution of the screening should be encouraged in specialized burn care. The multidimensional DSMS provides information on the affected domains; however, it is less often completed in severely injured patients. A more concise tool, including fewer to no separate domains, and thereby forming a less time-consuming instrument, might be considered for critically ill patients with a larger TBSA burned, especially since a substantial proportion of screened patients had a partially completed screening—an instrument that can be filled in at a glance might be preferred as the first screening. The DSMS frailty screening might then be used next, to determine which domains are likely to be problematic. Further prospective research is necessary to investigate this suggestion.

## Figures and Tables

**Table 1:** Baseline characteristics.

Characteristics	All, *n* = 515
Sex: woman (%)	260 (50.5)
Age, years (%)	
70–74	179 (34.8)
75–84	223 (43.3)
≥85	113 (21.9)
Year of admission (%)	
2012–2015	281 (54.6)
2016–2018	234 (45.4)
ASA score ^1^ (%)	
1–2	266 (54.0)
≥3	227 (46.0)
Aetiology ^1^ (%)	
Flame	192 (38.5)
Scald	106 (21.2)
Other thermal injury ^2^	122 (24.4)
Other skin-related diseases ^3^	79 (15.8)
TBSA (%)	
<5	285 (55.3)
5–19	168 (32.6)
≥20	62 (12.0)
ICU (%)	147 (28.5)
Revised Baux Score (median (P25–P75)) ^4^	88.0 (78.0–93.0)

All data are presented as median (P25–P75) or as *n* (%). ^1^ Missing values: ASA-score (*n* = 2); Aetiology (*n* = 16) ^2^ Including contact, fat, chemical, steam, electricity, other causes. ^3^ Including toxic epidermal necrolysis (TEN), traumatic lesions, necrotizing infection of the fascia and soft tissues. ^4^ Patients with thermal injury only (*n* = 436).

**Table 2:** Predictors for complete DSMS frailty screening.

	Frailty Screening		Logistic Regression	
Characteristics	Complete (*n* = 204)	None/Incomplete (*n* = 311)	Univariable	Multivariable
	*n* (%)	*n* (%)	OR (95% CI)	OR (95% CI)
Sex (%)				
Man	106 (52.0)	149 (47.9)	1	-
Woman	98 (48.0)	162 (52.1)	0.85 (0.60–1.21)	**-**
Age, years (%)				
70–74	67 (32.8)	112 (36.0)	1	-
75–84	91 (44.6)	132 (42.4)	1.15 (0.77–1.73)	-
≥85	46 (22.5)	67 (21.5)	1.15 (0.71–1.86)	-
Year of admission (%)				
2012–2015	92 (45.1)	189 (60.8)	1	1
2016–2018	112 (54.9)	122 (39.2)	**1.89 (1.32–2.70) ^1^**	**2.15 (1.42–3.25) ^1^**
Day of the week (%)				
Week	149 (73.0)	225 (72.3)	1	-
Weekend	55 (27.0)	86 (27.7)	0.97 (0.65–1.44)	-
ASA score ^2^ (%)				
1–2	115 (56.9)	151 (51.9)	1	-
≥3	87 (43.1)	140 (48.1)	0.82 (0.57–1.71)	-
TSBA (%)				
<5	124 (60.8)	161 (51.8)	1	1
5–20	72 (35.3)	96 (30.9)	0.97 (0.66–1.43)	0.90 (0.58–1.40)
>20	8 (3.9)	54 (17.4)	**0.19 (0.09–0.42) ^1^**	**0.12 (0.05–0.29) ^1^**
Revised Baux Score (median (P25–P75))	82.5(77.5–89.0)	85.5 (78.7–95.0)	**0.97 (0.96–0.98) ^1^**	**-**
ICU (%)				
No	165 (80.9)	203 (65.3)	1	-
Yes	39 (19.1)	108 (34.7)	**0.44 (0.29–0.68) ^1^**	**-**

^1^*p* < 0.05 (bold numbers) ^2^ *n* = 22 missing.

**Table 3:** Known-groups validity of the DSMS frailty screening.

	Positive Frailty Screening ^1^ (*n* = 100)	Negative Frailty Screening (*n* = 128)	Logistic Regression (Univariable)
	*n* (%)	*n* (%)	OR (95% CI)
Age (years)			
<75	22 (22.0)	57 (44.5)	1
≥75	78 (78.0)	71 (55.5)	2.85 (1.58–5.12) ^2^
ASA Score			
1–2	38 (38.4)	90 (70.9)	1
≥3	61 (61.6)	37 (29.1)	3.91 (2.24–6.82) ^2^

^1^ Positive frailty screening (≥2 domains scored positive) ^2^ *p* < 0.05.

**Table 4:** Predictive validity of the DSMS frailty screening.

	DSMS Frailty Screening		Logistic Regression (Unadjusted)	Logistic Regression (Adjusted ^3^)
	Positive Score (*n* = 100)	Negative Score (*n* = 128)	OR (95% CI)	OR (95% CI)
Length of stay (days)				
≤7	23 (23.0)	47 (36.7)	1	1
>7	77 (77.0)	81 (63.3)	**1.94 (1.08–3.50) ^2^**	**1.97 (1.05–3.70) ^2^**
Discharge location				
Home	53 (53.0)	104 (81.3)	1	1
Non-home ^1^	47 (47.0)	24 (18.8)	**3.84 (2.13–6.95) ^2^**	**3.20 (1.69–6.12) ^2^**
In-hospital mortality				
No	91 (91.0)	126 (98.4)	1	1
Yes	9 (9.0)	2 (1.6)	**6.23 (1.32–29.52) ^2^**	3.66 (0.68–19.86)

^1^ Including: nursing home, rehabilitation center, other hospitals and psychiatric ward ^2^ *p* < 0.05 (bold numbers) ^3^ Adjusted for age, TBSA, and inhalation injury.

**Table 5:** Association between frailty score, determinants, and adverse outcome.

	Logistic Regression (Univariable)
	OR (95% CI)
Demographic and injury characteristics	
Age (years)	**1.11 (1.06–1.17) ^1^**
Sex (female)	1.12 (0.67–1.90)
Burn size (TBSA%)	1.01 (0.98–1.04)
Inhalation injury	5.29 (0.58–48.00)
ASA score > 2	**3.91 (2.24–6.82) ^1^**
Adverse outcomes	
Length of stay (days)	**1.94 (1.08–3.50) ^1^**
Discharge location	**3.84 (2.13–6.95) ^1^**
In-hospital mortality	**6.23 (1.32–29.52) ^1^**

^1^*p ≤* 0.05 (bold).

**Table 6:** Factors independently associated with adverse outcomes.

	Length of Stay (>7 Days)	Non-Home Discharge Location	In-Hospital Mortality
	Log Regression (Multivariable) OR (95% CI)	Log Regression (Multivariable) OR (95% CI)	Log Regression (Multivariable) OR (95% CI)
Age (years)	0.97 (0.92–1.02)	1.05 (1.00–1.10) ^3^	**1.13 (1.00–1.27) ^1^**
Sex (female)	**2.87 (1.53–5.39) ^1^**	1.68 (0.88–3.18)	0.89 (0.23–3.44)
Burn size (TBSA%)	1.05 (1.00–1.10) ^2^	**1.08 (1.03–1.13) ^2^**	**1.06 (1.01–1.10) ^1^**
Inhalation injury	1.09 (0.11 (11.15)	1.23 (0.19–7.84)	2.50 (0.21–29.48)
ASA (>2)	1.01 (0.53–1.91)	**2.03 (1.06–3.90) ^2^**	3.45 (0.66–17.99)
DSMS score (≥2)	**2.09 (1.06–4.14) ^1^**	**2.80 (1.43–5.49) ^2^**	2.71 (0.48–15.47)

^1^*p* ≤ 0.05 (bold) ^2^ Original 95% CI 0.997–1.103, ^3^ Original 95% CI 0.995–1.103.

**Table 7:** DSMS frailty screening practice per domain (*n* = 515 patients).

DSMS Domain	Complete, *n* (%)	Incomplete, *n* (%)	Not Screened, *n* (%)
Risk of delirium	258 (50.1)	34 (6.6)	223 (43.3)
Risk of falling	255 (49.5)		260 (50.5)
Risk of ADL dependence	276 (53.6)	27 (5.2)	212 (41.2)
Risk of malnutrition	286 (55.5)	14 (2.7)	215 (41.7)

## Abbreviations

SD: Standard deviation; OR: Odds Ratio; TBSA: Total body surface area; 95% CI: 95% Confidence interval; Dutch Safety Management System.

## Appendix A

**Table A1:** Known-groups validity of the DSMS frailty screening (≥2).

	All Patients	Patients with Burn Injury Only
	Logistic Regression (Univariable)	Logistic Regression (Univariable)
	OR (95% CI)	OR (95% CI)
Age (years)		
<75	1	1
≥75	2.85 (1.58–5.12) ^1^	3.48 (1.80–6.74) ^1^
ASA Score		
1–2	1	1
≥3	3.91 (2.24–6.82) ^1^	3.73 (2.04–6.83) ^1^

^1^*p* < 0.05.

**Table A2:** Predictive validity of positive DSMS frailty screening (≥2).

	All Patients	Patients with Burn Injury Only
	Logistic Regression (Adjusted ^3^)	Logistic Regression (Adjusted ^3^)
	OR (95% CI)	OR (95% CI)
Length of stay (days)		
≤7	1	1
>7	**1.97 (1.05–3.70) ^2^**	**2.45 (1.20–4.98) ^2^**
Discharge location		
Home	1	1
Non-home ^1^	**3.20 (1.69–6.12) ^2^**	**3.16 (1.53–6.55) ^2^**
In-hospital mortality		
No	1	1
Yes	3.66 (0.68–19.86)	**10.38 (1.08–99.27)**

^1^ Including: nursing home, rehabilitation center, other hospitals and psychiatric ward ^2^ *p* < 0.05 (bold numbers) ^3^ Adjusted for age, TBSA, and inhalation injury.
